# Glucose-6-phosphate isomerase promotes the proliferation and inhibits the apoptosis in fibroblast-like synoviocytes in rheumatoid arthritis

**DOI:** 10.1186/s13075-015-0619-0

**Published:** 2015-04-14

**Authors:** Ming Zong, Tianbao Lu, Shasha Fan, Hui Zhang, Ruhan Gong, Lishan Sun, Zhiyan Fu, Lieying Fan

**Affiliations:** Department of Clinical Laboratory, Shanghai East Hospital, School of Medicine, Tong Ji University, 150 Ji Mo Road, Shanghai, 200120 People’s Republic of China

## Abstract

**Introduction:**

Fibroblast-like synoviocytes (FLS) play an important role in the pathogenesis of rheumatoid arthritis (RA). This study aimed to investigate the role of glucose 6-phosphate isomerase (GPI) in the proliferation of RA-FLS.

**Methods:**

The distribution of GPI in synovial tissues from RA and osteoarthritis (OA) patients was examined by immunohistochemical analysis. FLS were isolated and cultured, cellular GPI level was detected by real-time polymerase chain reaction (PCR) and Western blot analysis, and secreted GPI was detected by Western blot and enzyme-linked immunosorbent assay (ELISA). Doxorubicin (Adriamycin, ADR) was used to induce apoptosis. Cell proliferation was determined by MTS assay. Flow cytometry was used to detect cell cycle and apoptosis. Secreted pro-inflammatory cytokines were measured by ELISA.

**Results:**

GPI was abundant in RA-FLS and was an autocrine factor of FLS. The proliferation of both RA and OA FLS was increased after GPI overexpression, but was decreased after GPI knockdown. Meanwhile, exogenous GPI stimulated, while GPI antibody inhibited, FLS proliferation. GPI positively regulated its receptor glycoprotein 78 and promoted G1/S phase transition via extracellular regulated protein kinases activation and Cyclin D1 upregulation. GPI inhibited ADR-induced apoptosis accompanied by decreased Fas and increased Survivin in RA FLS. Furthermore, GPI increased the secretion of tumor necrosis factor-α and interleukin-1β by FLS.

**Conclusions:**

GPI plays a pathophysiologic role in RA by stimulating the proliferation, inhibiting the apoptosis, and increasing pro-inflammatory cytokine secretion of FLS.

## Introduction

Rheumatoid arthritis (RA) is a chronic inflammatory joint disease that eventually leads to the destruction of the joint architecture. Synovial hyperplasia is considered a hallmark of RA, in which fibroblast-like synoviocytes (FLS) and immune cells communicate in a unique inflammatory microenvironment. The hyperplasia of the synovial lining is largely composed of increased numbers of FLS and macrophages. Other inflammatory cells, such as mast cells, dendritic cells, macrophages, and lymphocytes, are also recruited and accumulate in the sub lining. As they accumulate to form pannus tissue, RA-FLS exhibit local tumor-like destructive and invasive characteristics. Moreover, FLS contribute to the inflammatory microenvironment through directly producing pro-inflammatory factors or indirectly activating or recruiting other immune cells [1,2]. Therefore, FLS play a critical role in RA pathogenesis, and targeting FLS might improve clinical outcomes of inflammatory arthritis without suppressing systemic immunity [3].

Glucose-6-phosphate isomerase (GPI; EC 5.3.1.9), also known as phosphoglucose isomerase and phosphohexose isomerase, catalyzes the interconversion of D-glucose-6-phosphate and D-fructose-6-phosphate, a crucial step in glycolysis and gluconeogenesis [4]. In addition to its enzymatic activity, GPI acts as a cytokine and growth factor in a wide variety of extracellular processes [5-8]. GPI has been identified as a motility factor: autocrine motility factor (AMF) [5], neuroleukin [7,8] or maturation factors [9]. AMF/GPI is a multifunctional cytokine that exhibits multifunctional growth factor-like activity via a unique cognate 78 kDa (glycoprotein 78, gp78) seven-transmembrane glycoprotein receptor (autocrine motility factor receptor, AMFR) [10]. Many studies have shown that AMF not only stimulates AMF-producing tumor cell motility in an autocrine manner, but also acts as a paracrine factor for vein endothelial cells. AMF induces angiogenesis by stimulating cell motility and up-regulating vascular endothelial growth factor receptor (VEGFR) expression [11]. Overexpression of AMF/GPI and AMFR has been found in a wide spectrum of malignancies, and is associated with cancer progression, metastasis and angiogenesis [12-16].

The autoantibodies against glucose-6-phosphate isomerase (anti-GPI Abs) were present in the K/BxN T-cell receptor (TCR)-transgenic inflammatory arthritis mouse model [17]. Furthermore, recombinant human GPI was shown to have the ability to induce chronic arthritis in the mice, which was major histocompatibility complex (MHC) associated and B-cell dependent [18]. The total GPI protein level, in both enzymatically active and inactive forms, was significantly higher in the sera of RA patients compared with patients with other immune-based or non-immune-based inflammatory arthritis [19]. We previously demonstrated that 76.1% of patients with RA, but not controls, had increased concentration of soluble GPI in their sera and synovial fluid (SF), and serum GPI concentration was higher in active RA patients than in non-active RA patients [20]. However, it remains unclear where excessive GPI comes from in RA joints, and whether it is associated with joint tissue pathological changes of RA. In this study, we aimed to characterize the features of autocrine GPI from RA-FLS, and clarify the role of GPI in the regulation of FLS proliferation and apoptosis.

## Methods

### Patients and controls

Synovial tissues were obtained from eight RA patients and eight osteoarthritis (OA) patients who underwent knee arthroscopic or replacement surgery at Shanghai East Hospital. Serum samples were taken before the surgery from all 16 patients. All the subjects fulfilled the 2010 American College of Rheumatology (ACR) criteria for the diagnosis of RA and OA [21]. Informed consent was obtained from all patients and the study protocol was approved by the Ethics Committee of Shanghai East Hospital (2012-df-043). There were no differences in ethnicity among the groups and no patient with RA or OA had tumor and renal disease. RA patients were divided into two groups according to serum GPI level: GPI (+) RA and GPI (−) RA, with 0.2 mg/L as the cut-off value.

### Isolation and culture of fibroblast-like synoviocytes

Synovial tissues were minced into pieces of 2 to 3 mm in size and spread on the bottom of cell culture flasks in RPMI 1640 medium (Life Technologies, Carlsbad, CA, USA) at 37°C for 6 hours. Next, the tissues were incubated with complete RPMI 1640 medium supplemented with 10% fetal calf serum in a humidified atmosphere containing 5% CO_2_. The medium was changed every three to five days and non-adherent tissue pieces were carefully removed. FLS were grown further over four to six passages. To characterize the cytological phenotype of synovial cultures, the third passage cells were stained with mouse monoclonal antibodies (mAb) to human fluorescein isothiocyanate (FITC)-CD14 and phycoerythrin (PE)-CD90 (eBioscience, San Diego, CA, USA), and showed 2.8% CD14 and 97.0% CD90 expression, as measured by flow cytometry. For cell treatment, FLS were treated with human GPI (Abcam product number: ab87625, endotoxin: <1 EU/μg protein, Cambridge, UK).

### RNA interference and plasmid construction

To design specific small interfering RNA (siRNA) targeting GPI and gp78 (named siGPI and sigp78), several sequences from the human *GPI* gene were selected using BLOCK-iT™ RNAi Designer (Life Technologies, Carlsbad, CA, USA). The target sequences for GPI siRNA (siGPI: 5′-CCATACGGAAGGGTCTGCATCACAATT-3′), gp78 siRNA (sigp78: 5′-GTCG GCACAAGAACTATCTTT-3′) and negative control siRNA (si-NC: 5′-TTCTCCGAA-CGTGTCACGT-3′) were synthesized by Genepharma Inc (Shanghai, China). SiRNA transfection was performed using Lipofectamine® 2000 (Invitrogen, Carlsbad, CA, USA). According to the sequence of GPI cDNA (NM_001184722.1), we designed the primers 5′-CGGAATTCCA TGGTAGCTCTCTGCAGCCT-3′ (forward) and 5′-CCCTCGAGGTTATTGGACTCTGG CCTCG C-3′ (reverse), and obtained the full length of GPI cDNA by PCR. GPI cDNA was inserted into pcDNA3.1-Flag (Invitrogen) vector and plasmid DNA transfection was performed using Lipofectamine -LTX (Invitrogen). PCI-Neo-gp78/JM20 (plasmid 13303) was purchased from Addgen (Teddington, UK).

### Immunohistochemical analysis

The tissues were fixed in 10% neutral buffered formalin and embedded in paraffin. Next, the tissues were cut into 5-μm thick sections, deparaffinized and rehydrated. The sections were then subjected to hematoxylin and eosin (H&E) or immunohistochemical staining. For immunostaining, the sections were heated at 95°C for 20 minutes with Dako Target Retrieval Solution (Dako, Copenhagen, Denmark). Sections were incubated with mouse anti-GPI mAb (1:500, Abcam Cambridge, UK) or mouse anti-gp78 mAb (1:100, Santa-Cruz Dallas, TX, USA) at 4°C overnight, and then incubated with second antibody (Envision™ Detection Kit, Dako) for 30 minutes at room temperature. Finally, the sections were visualized by using diaminobenzidine (DAB) substrate kit (Dako), according to the manufacturer’s instructions. Mouse serum was used instead of primary antibody as a negative control.

MTS assay. Cell proliferation was determined by using the CellTiter 96® Aqueous One Solution Cell Proliferation Assay kit (Promega, Beijing, China), according to the manufacturer’s instructions. Briefly, cells were plated at 1 × 10^3^ cells/well in 96-well plates and cultured for different periods. At the end of each period, 20 μl MTS was added to each well and then incubated at 37°C for 4 hours. Plates were read at 490 nm on a spectrophotometric plate reader (Bio-Rad, Hercules, CA, USA) with a reference wavelength at 650 nm. The index for stimulating cell proliferation was calculated as intervention group optical density (OD) value/blank control group OD value.

### Flow cytometry analysis of apoptosis and cell cycle

FLS were trypsinized and collected for the detection of apoptosis by using Annexin V-FITC Apoptosis Detection Kit (eBioscience). Briefly, FLS were washed twice with cold PBS and resuspended in 500 μl binding buffer (10 mM HEPES-NaOH, pH 7.4, 140 mM NaCl, 2.5 mM CaCl2) at a concentration of 1 × 10^6^ cells/ml. After the addition of 5 μl Annexin V-FITC solution and Propidium solution (PI, 2.5 μg/ml, Sigma-Aldrich, St. Louis, MO, USA), the cells were incubated for 15 minutes at room temperature and then analyzed by flow cytometer (Beckman Coulter, Fullerton, CA, USA). Moreover, cells in the logarithmic growth phase were prepared as a single cell suspension at 1 × 10^6^ cells/ml, fixed with ethanol and then cryopreserved. FLS were resuspended in 500 μl pre-cold PBS and incubated at 37°C for 30 minutes after the addition of RNase A solution. After the addition of 20 μl PI (50 μg/ml), the percentages of G1 phase, S phase and G2 phase cells were determined by flow cytometry. The index for apoptosis was calculated as the number of cells undergoing apoptosis/the number of total cells.

### Quantitative real-time polymerase chain reaction

Total RNA was extracted from FLS using TRIzol™ (Invitrogen), and reverse transcription was performed using first strand cDNA synthesis kit (TaKaRa, Dalian, China), according to the manufacturer’s instructions. Real-time PCR was performed using Premix Ex Taq SYBR Green PCR (TaKaRa), according to the manufacturer’s instructions, on an ABI PRISM 7300 (Applied Biosystems, Foster City, CA, USA). The sequences of the primers were as follows: GPI 5′-AGGCTGCTGCCACATA-AGGT-3′, 5′-AGCGTCGTGAGAGGTCACTTG-3′; gp78 5′-CCATCATCAG CGCCTAC CG-3′, 5′-AACCAGAGGCACCACATGAC-3′; Fas 5′-CCCACCTACGTA CTGGCCTA-3′, 5′ -CTTGGCCTTGGGTGTGTACT-3′; Bcl-2 5′-AGTTCGGTGG-GGTCATGTGTG-3′, 5′-CTTCAGAGACAGCCAGGAGAAATC-3′; Bax 5′-TTCTGACGGCAACTTCAACTG-3′, 5′-TGAGGAGGCTTGAGGAGTCTC- 3′; Survivin 5′- TGCCTGGCAGCCC TTTCTCA-3′, 5′-TGGCACGGCGCACTTTCTTC-3′; Caspase-3 5′-TGGAACAAATGG ACCTGTTGA-3′, 5′-TAATAACCAGGTGCTGTGGAGT-3′ and GAPDH 5′-TGACTT CAACAGCGACACCCA 3′, 5′ -CACCCTGTTGCTGTAG CCAAA −3′. GAPDH was used as the internal control.

### Western blot analysis

Approximately 2 × 10^6^ cells were lysed in lysis buffer and the lysates were centrifuged at 14,000 rpm for 15 minutes. The protein concentration in the supernatant was determined using the Bradford method (Bio-Rad). Protein samples were separated on 12% SDS-PAGE and then transferred onto nitrocellulose membranes (Amersham Pharmacia Biotech, Uppsala, Sweden). The membranes were incubated with antibody for GPI (Abcam), gp78 (Santa-Cruz), P-ERK1/2, ERK1/2, Cyclin D1 (Cell Signaling Technology, Danvers, MA, USA), Bax, Bcl2, Fas, Survivin, Cleaved-Caspase-3 or Caspase-3 (Abcam), then incubated with horseradish peroxidase-conjugated secondary antibody. All immunoreactive proteins were visualized with SuperSignals west Pico Chemiluminescent Substrate (Thermo Fisher Scientific, Rockford, IL, USA).

### Co-immunoprecipitation analysis

FLS were transfected with pcDNA3.1-GPI- Flag. The cell lysates were collected 48 hours later for co-immunoprecipitation (Co-IP) assay by using FLAG® Immunoprecipitation Kit (Sigma-Aldrich) according to the manufacturer’s instructions. The bound fragments were analyzed by Western blot analysis.

### Enzyme-linked immunosorbent assay

Human TNF-α, IL-1β, TGF-β and IL-6 levels were measured in the supernatant of FLS by using commercially available kits (R&D Systems China, Shanghai, China), according to the manufacturer’s instructions. Serum G6PI level was measured by using GPI enzyme-linked immunosorbent assay (ELISA) Kit (Beijia Biochemical, Shanghai, China). The biological reference interval of serum G6PI level was set to ≤0.2 mg/L, in accordance with the manufacturer’s instructions.

### Statistical analysis

For paired samples, Wilcoxon’s matched pairs signed rank test was used. For multiple related samples, the Friedman M test was used. Further pairwise comparisons were analyzed using a multiple related samples q test if appropriate. SPSS19.0 program package (SPSS Inc., Chicago, IL, USA) was used for all statistical analyses. A *P* value of less than 0.05 was considered statistically significant.

## Results

### Glucose 6-phosphate isomerase is abundant in fibroblast-like synoviocytes of glucose 6-phosphate isomerase (+) rheumatoid arthritis patients

**Figure 1:**
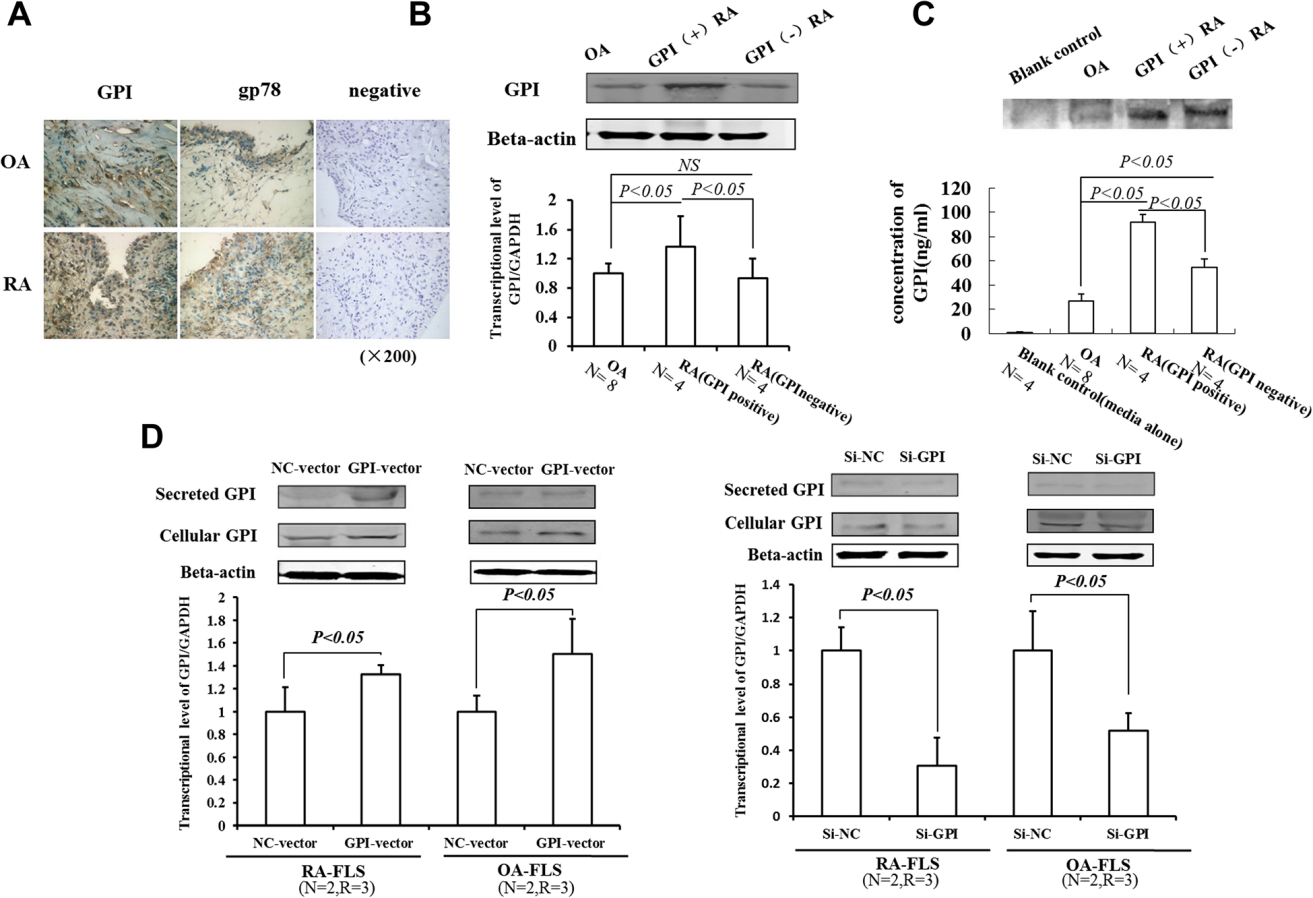
GPI is highly expressed and secreted in RA-FLS. **A**, Immunohistochemistry staining of GPI and GP78 in arthritic synovial tissues from RA and OA patients (OA serum GPI: 0.1 μg/ml; RA serum GPI: 1.8 μg/ml). Original magnification × 200. **B**, Western blot (top) and quantitative RT-PCR analysis (bottom) of GPI level in FLS. OA (n = 8, serum GPI concentration <0.2 μg/ml), GPI (+) RA (n = 4, serum GPI concentration = 1.3 ± 0.8 μg/ml), GPI (−) RA (n = 4, serum GPI concentration <0.2 μg/ml). Friedman’s M test and SNK-q test were used for statistical analysis. **C**, Western blot (top) and ELISA (bottom) analysis of GPI level in the supernatant of FLS. Friedman’s M test and SNK-q test were used for statistical analysis. **D**, Western blot (top) and quantitative RT-PCR analysis (bottom) of the levels of secreted and cellular GPI in FLS transfected with expression plasmids and siRNAs. Data were shown as mean ± SD. *P* values were determined by Wilcoxon signed-rank test. (N = number of individuals, R = number of replicates; GPI, glucose 6-phosphate isomerase; gp78, glycoprotein 78;RA, rheumatoid arthritis; OA, osteoarthritis; FLS, fibroblast-like synoviocytes; NC, negative control).

To compare the distribution of GPI and its receptor gp78 in synovial tissues from RA and OA patients, we performed immunohistochemical analysis and found that both GPI and gp78 were detected in the synoviocytes of RA and OA. GPI immunostaining was mainly localized in the cytoplasm and/or interstitial cell space and immunopositive cells had a diffuse distribution, while gp78 was mainly localized in the cytoplasm and was weak in the stroma (Figure 1A).

Furthermore, we detected the expression of GPI in FLS by Western blot and quantitative RT-PCR analysis. The results showed that GPI was present in FLS of both RA and OA. In addition, GPI level was higher in RA-FLS than in OA-FLS, and GPI level was increased in GPI (+) RA patients compared to GPI (−) RA patients (Figure 1B). These results demonstrate that GPI is abundant in RA-FLS, especially in GPI (+) RA patients.

### Glucose 6-phosphate isomerase is an autocrine factor of fibroblast-like synoviocytes

To test autocrine GPI/AMF in FLS, we detected secreted GPI in the culture supernatant of FLS by Western blot analysis (Figure 1C). Next, we transfected GPI siRNA and overexpression plasmid to RA-FLS, respectively. The results showed that the secreted GPI level was diminished in GPI knockdown FLS but was significantly increased in GPI overexpressing FLS, compared to control (Figure 1D). These results suggest that GPI is an autocrine factor of FLS.

### Glucose 6-phosphate isomerase promotes the proliferation of fibroblast-like synoviocytes

**Figure 2:**
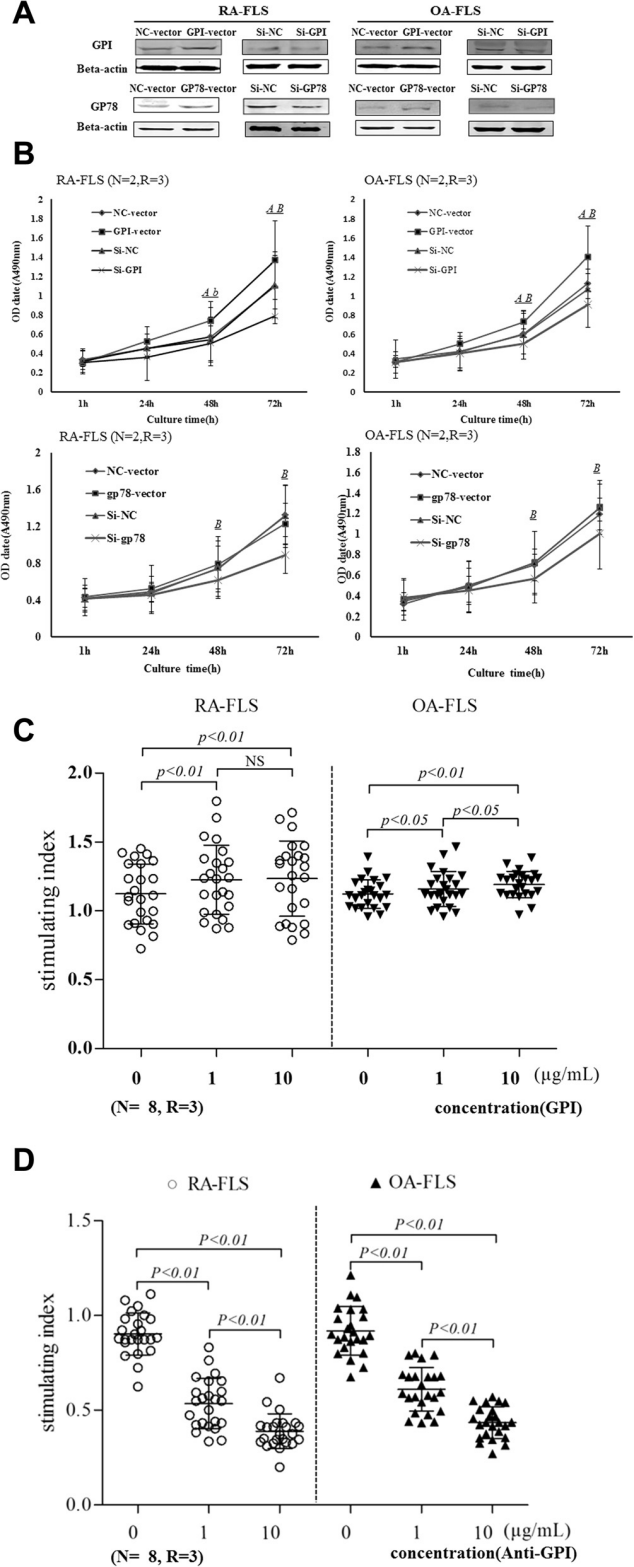
GPI promotes the proliferation of FLS isolated from RA or OA patients. **A**, FLS were transfected with expression plasmids and siRNAs. After 72 hours, the expression of GPI and GP78 was evaluated by Western blot analysis. **B**, The growth curve of FLS after GPI overexpression and knockdown (top). The growth curve of FLS after GP78 overexpression and knockdown (bottom). Cell proliferation capacity was determined by MTT assay every 24 hours. A(a) NC-vector versus GPI/gp78-vector, A: *P* <0.01, a: *P* <0.05; B(b) si-NC versus si-GPI/gp78, B: *P* <0.01, b: *P* <0.05. *P* values were determined by Wilcoxon signed-rank test. **C**, The proliferation of FLS after treatment with GPI (1, 10 μg/ml) for 48 hours. **D**, The proliferation of FLS after treatment with anti-GPI Ab (1, 10 μg/ml) for 48 hours. Friedman’s M test and SNK-q test were used for statistics. (N = number of individuals, R = number of replicates; GPI, glucose 6-phosphate isomerase; gp78, glycoprotein 78;RA, rheumatoid arthritis; OA, osteoarthritis; FLS, fibroblast-like synoviocytes; NC, negative control; OD, optical density).

To evaluate the pathological significance of GPI accumulation for RA progression, we modulated GPI and gp78 expression levels in RA-FLS by the transfection of the cells with GPI and gp78 siRNA duplexes or expression plasmids. As expected, the transfection of GPI and gp78 siRNA markedly reduced the protein levels of GPI and gp78, while the transfection of GPI and gp78 expression vector enhanced GPI and gp78 protein levels (Figure 2A). MTS assay showed that GPI overexpression dramatically increased the proliferation of both RA and OA FLS, whereas GPI knockdown reduced the proliferation of FLS. gp78 knockdown also reduced the proliferation of FLS, but gp78 overexpression had no significant effect on the proliferation of FLS compared to the control (Figure 2B).

To confirm these results, next we cultured RA-FLS and OA-FLS in growth medium in the presence of GPI or GPI antibody. GPI stimulated OA-FLS proliferation in a non-concentration-dependent manner, but promoted RA-FLS proliferation in a concentration-dependent manner (Figure 2C). On the contrary, GPI antibody showed inhibitory effects on the proliferation of RA-FLS and OA-FLS in a concentration-dependent manner (Figure 2D). Collectively, these results suggest that GPI promotes the proliferation of FLS.

### Glucose 6-phosphate isomerase regulates cell cycle of fibroblast-like synoviocytes

**Figure 3:**
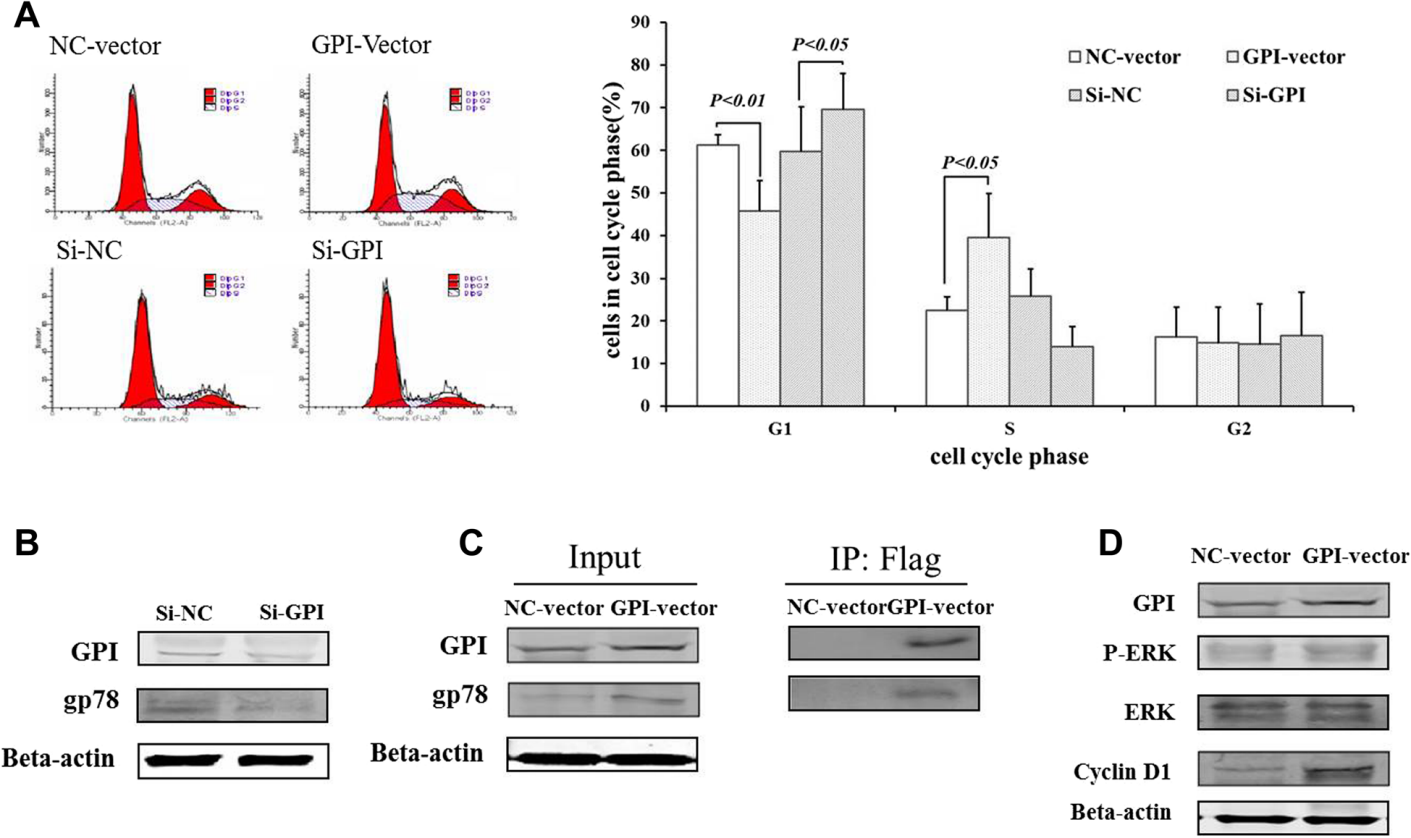
GPI regulates cell cycle progression of FLS. **A**, Flow cytometry of the cell cycle of RA-FLS after GPI overexpression and knockdown for 48 hours (left) and the percentages of the indicated cell subsets (right). **B**, Western blot analysis of the levels of GP78 in RA-FLS after GPI knockdown for 48 hours. **C**, Western blot analysis of the levels of GP78 in RA-FLS after GPI overexpression for 48 hours (left). The interaction between GPI and GP78 was determined by Co-IP analysis (right). **D**, Western blot analysis of the levels of P-ERK, ERK and Cyclin D1 in lysates of RA-FLS after GPI overexpression. β-actin was used as a loading control. *P* values were determined by Wilcoxon signed-rank test (GPI, glucose 6-phosphate isomerase; gp78, glycoprotein 78;RA, rheumatoid arthritis; OA, osteoarthritis; FLS, fibroblast-like synoviocytes; NC, negative control; ERK, extracellular regulated protein kinases).

Next, we examined the effects of GPI on the cell cycle of FLS. Flow cytometry analysis showed that the number of FLS was markedly lower in the G1 phrase, but was increased in the S phrase after transfection with GPI expression vector. On the contrary, GPI knockdown caused a stagnation of G1-S transition of FLS (Figure 3A). To investigate the mechanism by which GPI regulates the cell cycle of FLS, we found that knockdown of endogenous GPI led to the downregulation of its receptor gp78 (Figure 3B). Co-IP analysis showed a direct interaction between GPI and GP78 (Figure 3C). Moreover, we detected the levels of cell cycle regulatory proteins. Compared with the control group, the levels of P-ERK1/2 and Cyclin D1 were increased after GPI overexpression, while ERK1/2 level was not significantly changed (Figure 3D). These data indicated that GPI positively regulates its receptor gp78 and promotes G1/S phase transition via the activation of ERK and the upregulation of Cyclin D1 expression.

### Glucose 6-phosphate isomerase inhibits doxorubicin-induced apoptosis in rheumatoid arthritis-fibroblast-like synoviocytes

**Figure 4:**
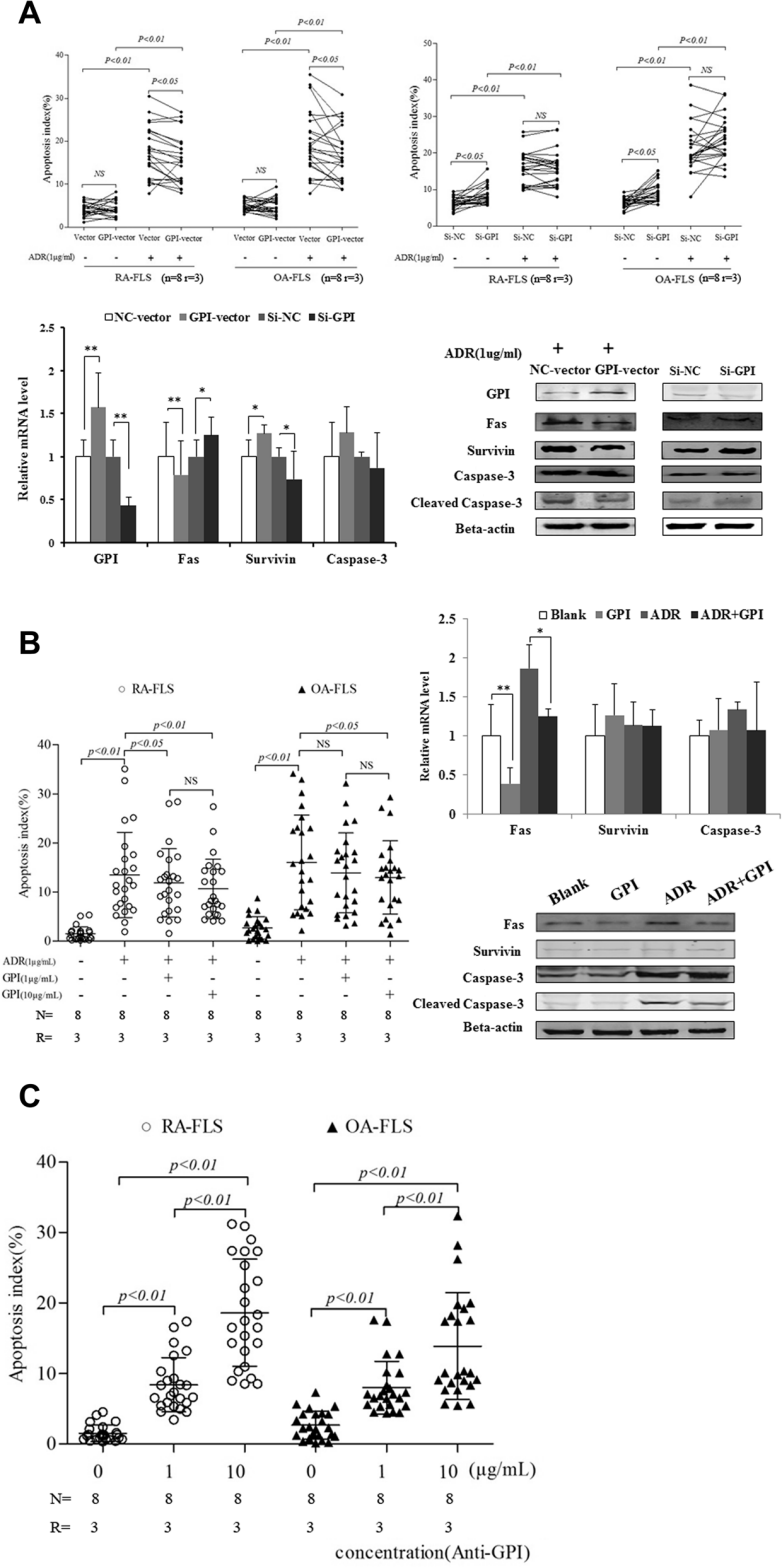
GPI inhibits the apoptosis of FLS isolated from RA or OA patients. **A**, FLS were transfected with GPI expression plasmid (top left) and siRNAs (top right), and 24 hours later were incubated in the absence or presence of 1 μg/ml of ADR for 48 hours. Cell apoptosis was detected by flow cytometry. Quantitative RT-PCR analysis of Fas, Survivin and Caspase-3 mRNA levels in RA-FLS after GPI overexpression and knockdown (bottom left). Western blot analysis of Fas, Survivin, Caspase-3 and Cleaved-Caspase-3 levels in RA-FLS after GPI overexpression and knockdown (bottom right). Friedman’s M test and SNK-q test were used for statistical analysis. **B**, FLS were pretreated with GPI, and 24 hours later were incubated in the absence or presence of 1 μg/ml of ADR for 48 hours. Cell apoptosis was detected by flow cytometry (left). Quantitative RT-PCR analysis of Fas, Survivin and Caspase-3 mRNA levels in RA-FLS after GPI or/and ADR treatment (right). Western blot analysis of Fas, Survivin, Caspase-3 and Cleaved-Caspase-3 levels in RA-FLS after GPI or/and ADR treatment (right). Friedman’s M test and SNK-q test were used for statistical analysis. **C**, FLS were pretreated with GPI antibody, and 24 hours later were incubated in the absence or presence of 1 μg/ml of ADR for 48 hours. Cell apoptosis was detected by flow cytometry. Friedman’s M test and SNK-q test were used for statistical analysis. (N = number of individuals, R = number of replicates, **P <0.01, *P <0.05).

Doxorubicin (Adriamycin, ADR) is a well-known apoptosis-inducing agent. To examine the effect of GPI on the apoptosis of FLS, we used ADR to treat RA-FLS. Flow cytometry analysis showed that GPI knockdown led to the apoptosis in FLS isolated from RA or OA, even without ADR treatment. When FLS were treated with ADR, the apoptosis ratios were increased in GPI knockdown and control cells, without significant difference between them. In contrast, GPI overexpression diminished the apoptosis of FLS treated with ADR (Figure 4A).

To further confirm the role of GPI in the apoptosis of FLS, we treated FLS from RA and OA patients with exogenous GPI, and detected the apoptosis of FLS by Annexin V-FITC/PI assay. We observed that GPI significantly decreased ADR-induced apoptosis of FLS isolated from RA (GPI at 1 μg/ml, *P* <0.05; GPI at 10 μg/ml, *P* <0.01). GPI also attenuated the apoptosis of FLS isolated from OA (GPI at 10 μg/ml, *P* <0.05) (Figure 4B). When we treated FLS from RA and OA patients with GPI antibody, GPI antibody consistently led to increased apoptosis of RA-FLS and OA-FLS in a concentration-dependent manner (Figure 4C).

To elucidate the molecular mechanism underlying GPI-attenuated apoptosis of FLS, we detected the expression of apoptosis-related proteins in RA-FLS. RT-PCR analysis showed that GPI overexpression led to a decreased Fas mRNA level and increased Survivin mRNA level, while GPI knockdown led to an increased Fas mRNA level and decreased Survivin mRNA level. Western blot analysis consistently showed that GPI overexpression led to decreased protein levels of Fas and Cleaved Caspase-3 and an increased Survivin protein level, while GPI knockdown led to increased protein levels of Fas and Cleaved Caspase-3 and a decreased Survivin protein level (Figure 4A).

Furthermore, we treated RA-FLS with GPI in the absence or presence of ADR. We observed that GPI significantly decreased the Fas mRNA level in both basic and ADR treatment conditions, while it had no significant effects on Survivin mRNA level. Western blot analysis confirmed that GPI decreased Fas protein level in RA-FLS in both basic and ADR treatment conditions, while it had no significant effects on Survivin protein level. In addition, GPI decreased ADR-induced cleavage of Caspase-3 in RA-FLS (Figure 4B). Taken together, these results indicated that the mechanism of GPI-attenuated apoptosis of FLS is associated with the inhibition of the Caspase pathway mediated by Fas.

### Glucose 6-phosphate isomerase modulates the secretion of cytokines by fibroblast-like synoviocytes

**Figure 5:**
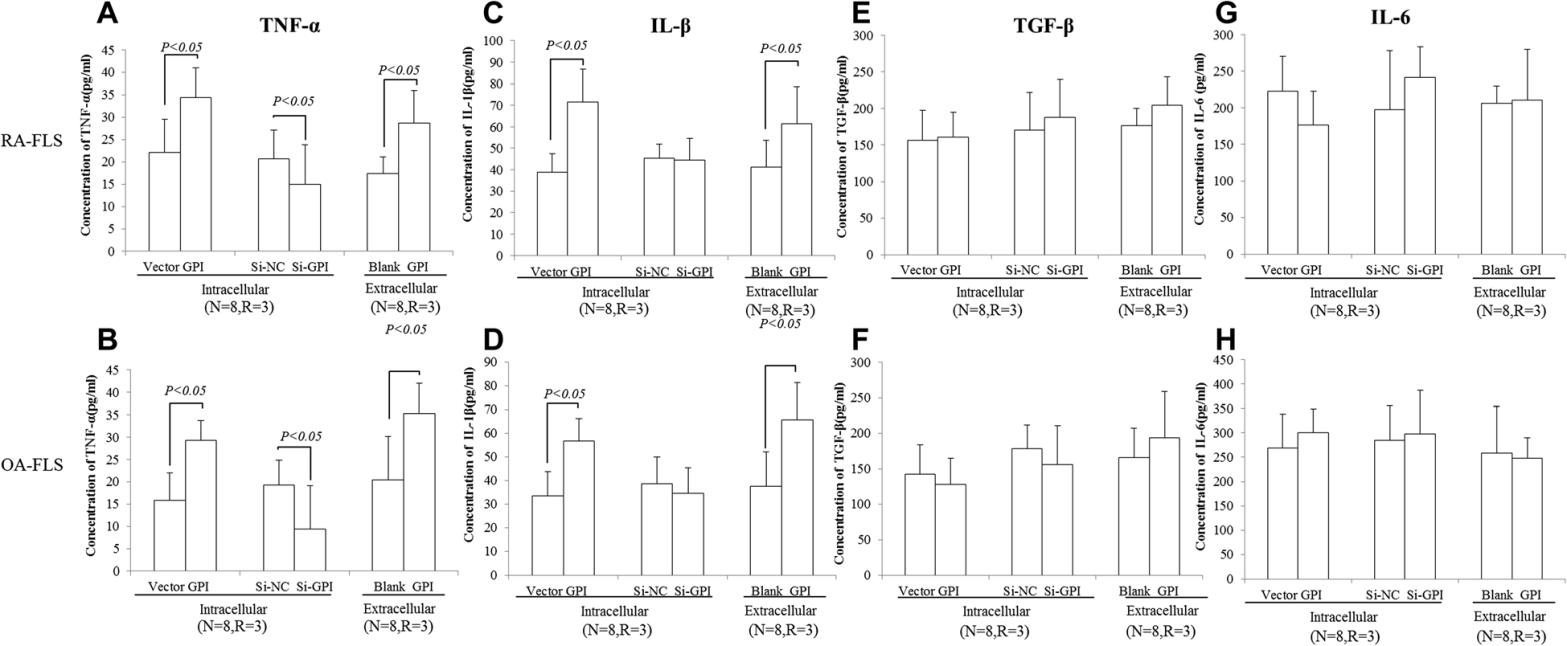
GPI modulates the secretion of TNF-α, IL-1β, TGF-β and IL-6 in FLS isolated from RA or OA patients. RA or OA FLS were transfected with GPI expression plasmids and siRNAs or treated with GPI at 1 μg/ml for 72 hours and the supernatants were collected for the analysis of TNF-α **(A and B)**, IL-1β **(C and D)**, TGF-β **(E and F)** and IL-6 **(G and H)** levels using ELISA. Data were shown as mean ± SD (N = 8, R = 3). *P* values were determined by Wilcoxon signed-rank test. (N = number of individuals, R = number of replicates).

Finally, we investigated the effect of GPI on the secretion of cytokines by FLS isolated from RA or OA patients. ELISA assay showed that the addition of GPI or the transfection of GPI expression vector increased the secretion of TNF-α and IL-1β by RA and OA FLS, while siRNA-mediated knockdown of GPI decreased the secretion of TNF-α by RA and OA FLS. In addition, the secretion of TGF-β and IL-6 by RA and OA FLS did not show any difference after GPI overexpression or knockdown (Figure 5). Collectively, these results suggest that GPI modulates the secretion of TNF-α and IL-1β by FLS.

## Discussion

It is generally accepted that GPI is closely associated with RA [22,23]. In our previous clinical study, we found that GPI could be a clinical marker for the diagnosis and therapy of RA [20]. However, the mechanism by which GPI contributes to the initiation and progression in RA remains elusive.

GPI has been shown to regulate the proliferation and survival of tumor cells and prevent stress-induced apoptosis and oxidative stress-induced cellular senescence in tumor cells [24-27]. In this study, we found that GPI is abundant in FLS isolated from patients with RA. To our knowledge, we demonstrate for the first time that GPI is also an autocrine factor of FLS. In addition, our results show that GPI significantly promoted FLS proliferation via enhancing G1-S transition due to activated ERK-Cyclin D1 signaling, and inhibited the apoptosis of FLS and augmented the secretion of pro-inflammatory cytokines by FLS. FLS are key players in the physiopathology of RA through the local secretion of pro-inflammatory cytokines, inflammation mediators and proteolysis enzymes that degrade components of extracellular matrix and destroy the joint structure [28]. Considering that the synovial tissue of RA is mainly composed of FLS, these effects of GPI would account for the pathogenicity of GPI in RA.

Several hypotheses have been proposed to explain elevated GPI antigen in the serum and SF of RA patients. The hypoxic microenvironment was considered to be one of the major factors contributing to GPI overexpression. Hypoxia enhances the expression of GPI in various cancer cells, including human pancreatic cancer cells and breast carcinoma cells, accompanied by increased motility of cancer cells [29-32]. Thus it is proposed that GPI may be upregulated due to hypoxia in the rheumatoid joint in RA [33]. Immunohistochemistry analysis demonstrated that distinct cells at the synovial surface lining of inflamed RA but not OA joints contained a high concentration of GPI [19]. However, there is still no direct evidence that synovial fibroblasts are capable of secreting GPI. In this study, we demonstrated that RA-FLS, especially isolated from GPI (+) RA patients, overexpressed and secreted GPI protein. Previous studies have suggested that abnormally elevated GPI in the SF of RA patients may come from T cells and the infiltrating inflammatory cells in the synovium [9,20]. In addition, the possibility that the diffusion of GPI from plasma into SF cannot be excluded. However, our results show that autocrine GPI in FLS contributes to the elevated level of GPI antigen in SF.

Since GPI is an autocrine factor of FLS, we further investigated how GPI contributes to the pathogenesis of RA. GPI is the second enzyme in the glycolytic pathway and catalyzes the interconversion of glucose 6-phosphate and fructose 6-phosphate during glycolysis and gluconeogenesis. It is highly conserved in bacteria and eukaryotes [34]. Meanwhile, GPI has been found to function both as a cytokine and as a growth factor, to regulate cell migration, proliferation, apoptosis and cellular senescence. The levels of GPI and its cell surface receptor gp78 are associated with the pathologic stage, grade and prognosis of tumors [35-37]. Suppression of GPI led to a contact-dependent inhibition of cell growth and inhibited the ability of cells to form tumor mass [25,38]. In this study, we found that both endogenous GPI and exogenously added GPI promoted FLS proliferation, while suppression of GPI and gp78 led to the inhibition of cell growth. Moreover, GPI antibody decreased FLS proliferation in a concentration-dependent manner. To better investigate the effect of GPI on FLS proliferation, we assessed cell cycle progression, and found that G1-S transition was blocked after GPI knockdown. Taken together, our results suggest that GPI promotes cell cycle progression and proliferation of FLS.

**Figure 6:**
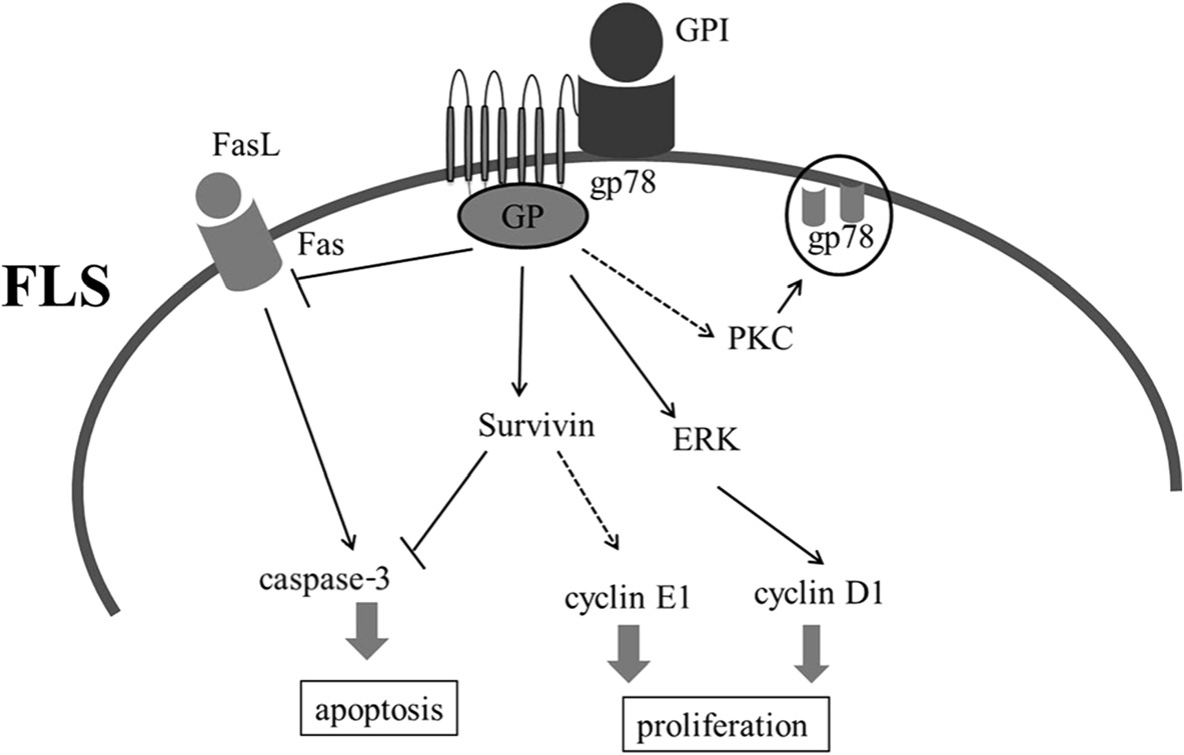
Schematic model for the role of GPI in the regulation of RA-FLS proliferation and apoptosis. ERK, extracellular signal-regulated kinase; Fas, factor-associated suicide; FasL, factor-associated suicide ligand; FLS, fibroblast-like synoviocytes; GPI, glucose 6-phosphate isomerase; gp78, glycoprotein 78; PKC, protein kinase C.

GPI stimulates cell motility and growth via a receptor-mediated signaling pathway involving receptor phosphorylation (a pertussis toxin-sensitive G-protein), inositol phosphate production, protein kinase C activation and enhanced production of a metabolite of arachidonic acid (12 (S)-hydroxyeicosatetraenoic acid cells) [39]. Our data indicate that gp78 expression was upregulated by GPI and there was a direct interaction between GPI and GP78. Moreover, we found that the upregulation of GPI markedly activated ERK signaling and increase the expression of Cyclin D1. ERK protein is involved in several regulatory pathways that regulate cell proliferation and survival [40]. Cyclin D1/CDK4/CDK6 is a central mediator in the transition from G1 to S phase. CDK4 and CDK6 form complexes with D-type Cyclins (D1, D2 and D3) and become active through the phosphorylation by CDK7/Cyclin H/MATer1 (CDK activating kinase; CAK) [41]. Cyclin D1 expression can be regulated positively by mitogen-activated protein kinases (MAPKs) pathway [42]. gp78 is a G-protein coupled receptor (GPCR) that can active ERK cascade reaction. Collectively, we hypothesized that GPI stimulates cell cycle progression of FLS via a receptor-mediated signaling pathway that involves gp78 overexpression, GPCR-ERK signaling activation and Cyclin D1 upregulation (Figure 6).

The overgrowth of FLS in RA is also likely due to an imbalance between cell proliferation, survival and death. In this study, we proved that GPI could inhibit the apoptosis of FLS, which may promote their survival and participation in the pathogenic process. Apoptosis is tightly regulated by anti- or pro-apoptotic molecules. The Fas/Caspase signaling pathway is one of the most critical pathways of apoptosis [43]. In the present study, we showed that endogenous GPI significantly inhibited the expression of Fas and the cleavage of Caspase-3, and promoted the expression of Survivin in FLS. Survivin is an important inhibitor of apoptosis that is undetectable in terminally differentiated adult tissues, but is overexpressed in cancer [44]. Survivin forms complexes, leading to the release of p21/wafl/cipl for Caspase inactivation [45,46]. Therefore, these results demonstrate that GPI-regulated apoptosis may be due to the Fas/Caspase and Survivin-dependent signaling pathway, and further show that GPI may play an important role in the promotion of rheumatoid synovial hyperplasia via the inhibition of FLS apoptosis. However, the expression of Survivin did not change in FLS treated by exogenously added GPI. Further investigation is necessary to elucidate the different effects of endogenous and exogenous GPI on Survivin expression.

It has previously been shown that the level of C1q/GPI-containing immune complexes (C1q/GPI-CIC) in the SF of RA patients was significantly higher than that of the non-RA group, but anti-GPI antibodies did not exhibit a significant difference [20]. Furthermore, the transfer of purified GPI-specific autoantibodies from K/BxN mice was able to induce arthritis in DBA/1 mice with features similar (but not identical) to those of human RA, and the deposition of GPI-CIC and activated C3 was found on the articular cartilage surface [22]. These studies indicated that GPI and anti-GPI antibodies might activate the complement pathway and induce arthritis. In our *in vitro* cell study, monoclonal anti-GPI antibody blocked extracellular GPI, leading to the decreased growth and increased apoptosis of FLS. These data showed that anti-GPI antibody played a dual role in GPI-mediated arthritis. Monoclonal anti-GPI antibody could block receptor-mediated signal and induce the apoptosis of FLS. On the other hand, anti-GPI antibody may play a positive role in the progression of arthritis.

Cytokines are known to play key roles as the mediators of RA progression. In particular, TNF-α and IL-1β act at the upstream of cytokine cascade to induce an excessive inflammatory reaction *in vivo* [47]. TNF-α appears to play important role in triggering events leading to inflammation both locally and systemically, whereas IL-1β is involved in cartilage and bone destruction [48]. In the present study we demonstrated that GPI promoted the secretion of TNF-α and IL-1β by FLS isolated from RA and OA. This further expands the importance of the pathophysiologic role of GPI in RA.

## Conclusions

Taken together, our findings demonstrate that GPI plays a potential pathophysiologic role in RA through the promotion of proliferation via a receptor-mediated signaling pathway, the stimulation of the secretion of inflammatory cytokines and the suppression of FLS apoptosis. Moreover, the elevated expression of GPI and the autocrine secretion of GPI by FLS are important aspects of RA pathology. These data strongly suggest that GPI is a promising therapeutic target for RA.

## Abbreviations

ADR: Adriamycin
AMF: Autocrine motility factor
FLS: Fibroblast-like synoviocytes
GPI: Glucose 6-phosphate isomerase
OA: Osteoarthritis
RA: Rheumatoid arthritis
SDS-PAGE: Sodium dodecyl sulphate polyacrylamide gel electrophoresis
siRNAs: small interfering RNAs
TNF-α: tumor necrosis factor-α
IL-1β: interleukin-1β
TGF-β: interleukin-1; transforming growth factor-β
IL-6:VEGFR: vascular endothelial growth factor receptor
ELISA: Enzyme-linked immunosorbent
GPCR: G-protein coupled receptor
ERK: extracellular regulated protein kinases
